# Sonographic Fetal Death in Goats in Khartoum State, Sudan: A Cross-Sectional Study

**DOI:** 10.1155/2019/1278389

**Published:** 2019-02-10

**Authors:** A. S. Aban, A. M. Almubarak, N. A. Abass, M. E. Badawi, M. T. Ibrahim, R. M. Abdelghafar

**Affiliations:** ^1^Faculty of Veterinary Medicine, Upper Nile University, Malakal, South Sudan; ^2^College of Veterinary Medicine, Sudan University of Science and Technology, P. O. Box 204, Hilat Kuku, Khartoum North, Sudan; ^3^College of Science and Technology of Animal Production, Sudan University of Science and Technology, P. O. Box 204, Hilat Kuku, Khartoum North, Sudan

## Abstract

In order to determine the prevalence of fetal mortality and investigate hypothesized risk factors associated with its occurrence in goats, 962 female goats were studied using ultrasound. To diagnose pregnancy and to identify dead fetuses, ultrasound scanning was conducted using real-time machines equipped with a transabdominal curvilinear probe. A questionnaire was supplied for collection of signalment and sampling data. Ultrasound examination revealed that out of 962 female goats, 431 (44.8%) goats were diagnosed as nonpregnant, 88 (9.14%) were pseudopregnant, 4 (0.42%) were diagnosed as having pyometra, and 439 were diagnosed as pregnant (45.63%). Of the 439 pregnant goats, 36 were diagnosed as bearing dead fetuses (8.2%). Season of the year, locality, breed of the dam, age of the dam, parity number, breed of the buck, and feeding type were all found not to be significantly associated with fetal death. It is concluded that ultrasound is a reliable method for diagnosis of fetal death and documenting the prevalence of its occurrence in goats.

## 1. Introduction

According to the food and agriculture organization (FAO, 2014) of the United Nations, there were an estimated 962 million goats in the world [1]. “Today, goats are among the most important livestock species in developing countries and they are of significant socioeconomic, nutritional and cultural importance in small holder farming system" [2].

In Sudan, there are about 31 million head of goats [3]. Goats are very important in alleviation of poverty and considered as “poor men cows” in rural communities as they provide job opportunities for women and children [4, 5]. Early and precise diagnosis of pregnancy, determination of fetal number, and gestational age in goats are crucial for improving livestock economy [6–10].

Fetal mortality contributes to large economic loss because it leads to loss of offspring and increases open periods [11, 12]. The first signs of prenatal death are absence of heartbeats and fetal movements [13, 14]. Ultrasonography of fetal fluids and placenta can be helpful in identifying dead or retarded fetuses; missing and cloudy fetal fluids as well as unclearly imaged placentomes suggest fetal death [11, 15]. Lack of echogenicity and proper amount of the amniotic fluid for the gestational age and normal fetal posture are signs of a healthy fetus [16]. Fetal measurements less than dated gestational age may indicate fetal death [11]. Ultrasound is one of the most extensively used imaging methods in veterinary medicine and has a very useful impact in diagnosis worldwide. It is considered a method of choice for detection of intrauterine fetal mortality [17].

In Sudan, there are only two published reports regarding diagnosis of fetal mortality using ultrasound technique [13, 14]. The aims of the present work were to study the prevalence of fetal mortality and to investigate the risk factors associated with its occurrence in goats in Khartoum State using ultrasound.

## 2. Materials and Methods

This study was conducted with the approval of the Research Committee of the College of Veterinary Medicine, Sudan University of Science and Technology. A cross-sectional study was performed on 962 female goats of different breeds (Saanen, Sannen crosses, Damascus, and Local) from different localities of Khartoum State (Khartoum, Um Bada, Bahri, Sharq El Nile, Karari, and Um Durman) through 2015-2016.

The sample size was calculated according to [18] (where P = prevalence; Q = 1-P; L^2^ = allowable error), with 41.28 % prevalence reported by [19] and 5% allowable error.

A well-prepared questionnaire was used to collect information about risk factors expected to have a relationship with fetal death. Season of the year, locality, breed of the dam, age of the dam, parity number, feeding type, breed of the buck, oestrous type, and rearing system were all registered as assumed risk factors for fetal death. Data concerning the owners of the goats (name, address, and telephone number) were also recorded.

Ultrasonographic transabdominal approach was performed using a real-time scanner (Pie medical esaote, Aquila vet, Holland) equipped with a switchable frequency (3.5-5) MHz convex probe, and Aloka SSD 500 Japan equipped with a 3.5 MHz curvilinear probe. The animals were restrained by two persons while they were turned on their backs on a table. The area used for scanning [20] was shaved appropriately using manual clippers. The probe was held in longitudinal, transverse, and oblique positions to ensure accurate diagnosis. Liberal amount of ultrasonic gel (Aquasonic-Turkey) was applied to the area prior to the scanning.

Statistical Packages for Social Sciences (SPSS) version 22 was used for the analysis. Univariate analysis using chi-square test (*χ*^2^) was done and a 5% level is considered significant.

## 3. Results and Discussions

Based on ultrasonographic scanning, out of 962 female goats, 439 were diagnosed as pregnant (45.6%). Animals were considered pregnant when a fluid-filled gestational sac with fetal parts and placentomes were documented. Of the 439 pregnant goats, 36 (8.2%) were diagnosed as bearing dead fetuses, which was determined when there was an absence of fetal heartbeats and/or movements. The remaining goats studied (523) included 431(44.8%) that were found not to be pregnant, 88 (9.14%) with a pseudopregnancy, and 4 (0.42%) with a pyometra. Pseudopregnancy was determined when an anechoic/hypoechoic compartmentalized fluid-filled uterus with absence of placentomes and fetal parts was found. Pyometra was characterized by a compartmentalized uterus that was fluid-filled hypoechoic with echogenic spots and debris.

The prevalence of fetal death in goats in Khartoum State was found to be 8.2 %. Season of the year (**χ**^2^ =4.873,* P* value 0.087), locality (**χ**^2^ =4.398,* P* value = 0.494), breed of the dam (**χ**^2^ =7.147,* P* value = 0.067), age of the dam (**χ**^2^ =2.566,* P* value = 0.272), feeding type (**χ**^2^ =0.728,* P* value = 0.394), parity number (**χ**^2^ =3.024,* P* value = 0.221), and breed of the buck (**χ**^2^ =0.560,* P* value = 0.756) were all found not to be significantly associated with fetal death in the present study (Table 1). Type of estrus and rearing system were not submitted for statistical analysis because all animals examined were naturally mated and reared under closed system.

Real-time ultrasonography is a method of choice for detecting fetal death throughout gestational period in goats. In the present study, transabdominal approach was used for diagnosis of fetal death and only single ultrasound scanning was performed to confirm the death of the fetus which is in agreement with [21]. Absence of fetal movements and heartbeats were the first documented signs of fetal death in the current study.

The recorded prevalence of fetal death (8.2%) was found lower than [19] who identified embryonic and fetal death at a rate of 41.28% when using both transrectal and transabdominal approaches during different stages of pregnancy. However, in the current study, all goats were examined using transabdominal approach and only fetal death was identified.

No association was found between the breed of the dam and fetal death. The high percentage of fetal death was reported from Saanen goats (30/36) and this could be due to the fact that 273 out of 439 (62%) of the pregnant goats belonged to the Saanen, a temperate breed which was imported to live in a tropical regions.

No significant association was found between season of the year and fetal death, although the dry summer season seemed to have a higher rate of fetal deaths (13.4%) relative to winter (6.7%) and fall (6.2%). This slightly higher rate of fetal deaths in the dry summer could be attributed to high temperatures, which decrease feed intake and increase heat stress. The lower percentage of fetal death reported from the fall season is likely due to this time being the breeding season for goats. Our results agree with [22] who reported high prevalence of pregnancy wastage in dry season.

A significant association was not found between locality and fetal death. This could most likely be due to the same environmental condition between different localities of Khartoum State. Additionally, although no significant association was found between parity number and fetal death which is in agreement with [19], some noticeable differences in fetal death rates were observed. A high percentage of fetal death was observed in multiparous goats (10.28%) compared to nulliparous (6.7%) and primiparous (3.77%). In multiparous goats, bacteria may gain access to the uterus during previous parturitions and oestrous cycles, which could lead to endometritis and pyometra. Infections may affect embryos negatively and lead to high incidence of fetal mortality [21, 23, 24].

No significant association was found between age of the dam and fetal death. The higher percentage (11.8%) of fetal death was found in the age group more than 4 years of age. References [21, 23, 24] stated that higher incidence of fetal death in animals over 6 years may be due to deterioration of the uterine environment in elderly animals, resulting in inauspicious medium for continuance of pregnancy.

## 4. Conclusions

It is concluded that real-time ultrasonography is an excellent tool for documenting the prevalence of fetal death. Further studies to identify other potential risk factors leading to fetal death should be conducted.

## Figures and Tables

**Table 1:** Univariate analysis of associated risk factors with fetal death in 439 pregnant goats using chi-square test.

Risk factors	No. examined	No. positive	%	*χ* ^2^	df	P value
*Season of the year*						
Winter	180	12	6.7			
Dry summer	115	15	13.04	4.873	2	.087
Wet summer	144	9	6.3			
*Locality*						
Khartoum	84	10	11.9			
Um-Badah	46	5	10.9			
Bahri	47	3	6.38	4.398	5	.494
Sharq El Nile	126	10	7.94			
Karari	121	6	5.00			
Um-Durman	15	2	13.3			
*∗Breed of the dam*						
Saanen	273	30	11.0			
Saanen crosses	76	3	3.94	7.147	3	.067
Damascus	44	1	2.27			
Local breed	40	2	5.0			
*∗Age of the dam*						
0.5-2 year	185	11	5.94			
> 2-4 years	148	15	10.13	2.566	2	0.277
> 4-6 years	34	4	11.76			
*∗Parity number*						
Nulliparous	119	8	6.72			
Primiparous	53	2	3.77	3.024	2	0.221
Multiparous	243	25	10.28			
*∗Breed of buck*						
Saanen	321	29	9.03			
Damascus	33	2	6.06	0.560	2	0.756
Local breed	18	1	5.55			
*Feeding type*						
Roughages	8	0	0.00	0.728	1	0.394
mixed	431	36	8.35			

*df = degree of freedom, χ* ^*2*^ *= chi square, P value = probability value*

*∗* numbers less than the total (439) is due to unavailable data.

## Data Availability

The data used to support the findings of this study are available from the corresponding author upon request.
